# A relational approach to improving interprofessional teamwork in post-partum haemorrhage (PPH)

**DOI:** 10.1186/s12913-022-08463-8

**Published:** 2022-09-01

**Authors:** Victoria Brazil, Darren McLean, Belinda Lowe, Lada Kordich, Deborah Cullen, Victoria De Araujo, Talia Eldridge, Eve Purdy

**Affiliations:** 1 Bond University, Gold Coast Health, Gold Coast, Australia; 2 Gold Coast Hospital and Health Service, Gold Coast, Australia

**Keywords:** Healthcare teamwork, Interprofessional collaboration, Post partum haemorrhage, Simulation

## Abstract

**Background:**

Post-partum haemorrhage (PPH) is an obstetric emergency that requires effective teamwork under complex conditions. We explored healthcare team performance for women who suffered a PPH, focusing on relationships and culture as critical influences on teamwork behaviours and outcomes.

**Methods:**

In collaboration with clinical teams, we implemented structural, process and relational interventions to improve teamwork in PPH cases. We were guided by the conceptual framework of Relational Coordination and used a mixed methods approach to data collection and analysis. We employed translational simulation as a central, but not singular, technique for enabling exploration and improvement. Key themes were identified from surveys, focus groups, simulation sessions, interviews, and personal communications over a 12-month period.

**Results:**

Four overarching themes were identified: 1) Teamwork, clear roles and identified leadership are critical. 2) Relational factors powerfully underpin teamwork behaviours—shared goals, shared knowledge, and mutual respect. 3) Conflict and poor relationships can and should be actively explored and addressed to improve performance. 4) Simulation supports improved team performance through multifaceted mechanisms.

One year after the project commenced, significant progress had been made in relationships and systems. Clinical outcomes have improved; despite unprecedented increase in labour ward activity, there has not been any increase in large PPHs.

**Conclusions:**

Teamwork, relationships, and the context of care can be actively shaped in partnership with clinicians to support high performance in maternity care. We present our multifaceted approach as a guide for leaders and clinicians in maternity teams, and as an exemplar for others enacting quality improvement in healthcare.

## Background

High quality care for women who suffer a postpartum haemorrhage (PPH) requires effective teamwork under complex conditions. Despite efforts to train teams and improve systems, interprofessional teamwork remains difficult. Complexity and relationships remain unaddressed in most teamwork courses, and in our predominantly linear approaches to system improvement. Given that PPH is the leading cause of maternal morbidity and mortality worldwide, [1] it is a natural focal point for exploring the context, relationships and culture within a maternity unit. Without a relationship-based approach, current efforts to improve teams and systems may fail to improve maternity care.

The incidence of PPH is increasing; a review of women birthing in Victoria from 2009–2013 found over 20% had a postpartum haemorrhage [2]. The reasons are not clear, and hence not easily targeted in healthcare improvement efforts. Changed characteristics of the maternal population such as increasing age, caesarean section rates and obstetric interventions may be contributing factors [2]. Without obvious targets for prevention, efforts to improve early recognition and effective management are critical.

The relationships, dynamics, and coordination between teams caring for women who suffer a PPH is complex. Time critical and potentially lifesaving action is required from clinical staff drawn from midwifery, obstetrics, anaesthesia, operating theatre and support teams including blood bank, porterage and hospital switchboard. In exploring and describing the features of a ‘very safe maternity unit’, Liberati et al. identified six core mechanisms—collective competence; insistence on technical proficiency; monitoring, coordination, and distributed cognition; clearly articulated and constantly reinforced standards of practice, behaviour, and ethics; monitoring multiple sources of intelligence about the unit's state of safety; and a highly intentional approach to safety and improvement [3, 4]. This landmark paper underpins the critical importance of exploring and shaping the *context* in which teams work, and the values that underpin these key behaviours. The authors caution against reliance on an ‘intervention logic’ in which quality improvement initiatives are done *to* staff, rather than *with* them. In a similar vein, Geary et al. describe a structured programme focused on improving culture in maternity teams that “placed quality and process improvement in the hands of the interprofessional frontline team” and measurably decreased the number and cost of reportable harm events [5]. Given this compelling argument, we sought ways to translate these ‘context and values’ to a structured method for exploration and intervention of the work of teams caring for women with PPH.

**Fig. 1:**
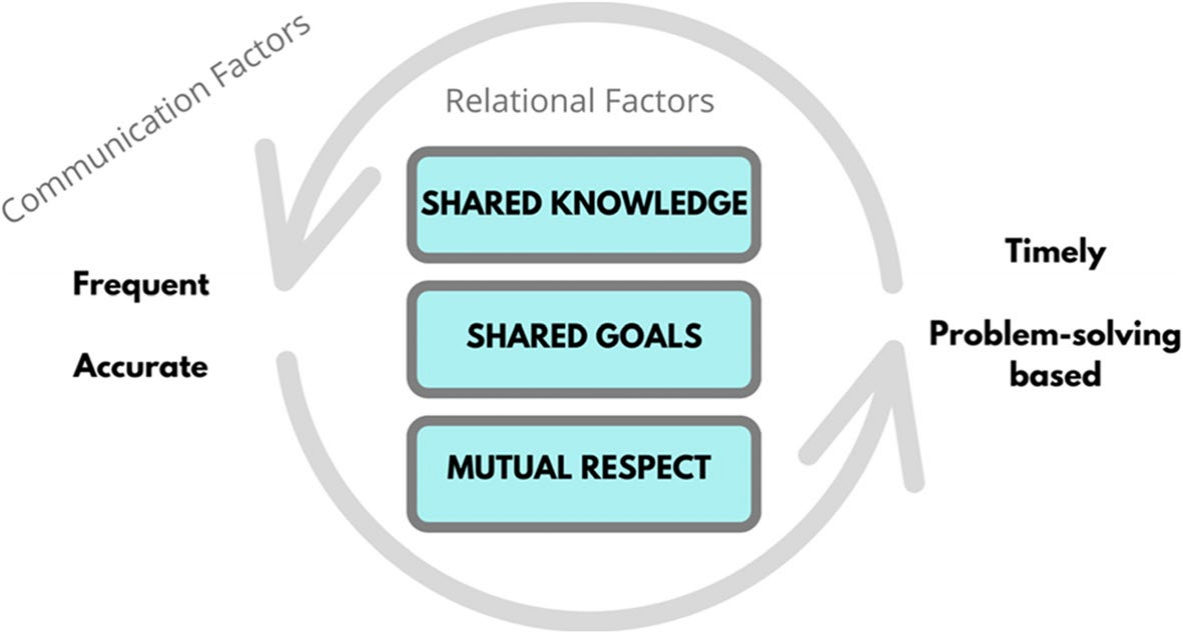
Relational Coordination framework—adapted from Gittell et al [7].

Relational Coordination (RC) [6] is one theory that allows for in-depth interrogation of relationships between groups involved in a focal work process (such as PPH); making it a logical framework for further understanding the maternity care context and a useful scaffold for shaping it. Relational coordination specifies three attributes of relationships that support the highest levels of coordination and performance: Shared goals that transcend participants’ specific functional goals, shared knowledge that enables participants to see how their specific tasks interrelate with the whole process, and mutual respect that enables participants to overcome the status barriers that might otherwise prevent them from seeing and taking account of the work of others. These three relational dimensions are reinforced by specific dimensions of communication that support coordination and high performance, namely frequency, timeliness, accuracy and, when problems arise, a focus on problem-solving rather than blaming [7] (Fig. 1).

Guided by the conceptual framework of Relational Coordination and using translational simulation [8] as a diagnostic and testing tool, we sought to concurrently study and support teams improving the care of women with PPH at Gold Coast University Hospital (GCUH). We hypothesised that improvement strategies based on education, training and systems changes require complementary focus on the relationships and culture within teams.

## Methods

We used a collaborative action research approach – a “process of deep inquiry into professional interactions with others in service of moving towards an envisioned future” [9]. It is done with people in a social context and involves probing multiple understanding of complex social systems, with a process of data sharing and knowledge construction. The subject(s) of action research are not people, rather the actions taken, the resulting changes, and the transformation in thinking, acting, and feeling by the persons enacting the change. The process of change is social.

### Setting

GCUH provides birthing services to the Gold Coast community and employs 136 midwives and 53 doctors who provide maternity care for women. There are over 5600 births per year with a baseline PPH (> 500) rate of ~ 24% and massive PPH (> 1.5L) rate of ~ 2%.

Our institution has an active Simulation Service, employing ‘translational simulation’ to directly target improving healthcare systems – through exploring work environments and the people in them, and through testing new or revised clinical pathways or processes [8]. Previous simulation programs at GCUH have engaged clinical teams from trauma care, emergency medicine, operating theatres and mental health in quality improvement and organisational learning [10, 11], and in shaping the values, beliefs, and practices of healthcare teams [12, 13].

Before our study started, GCUH maternity teams already employed simulation in full day courses, for in situ simulations in the ward environment, and in multidisciplinary simulations involving broader team interfaces. Full day courses—Simulated Emergency Response Training (SMART) or Practical Obstetric Multi-Professional Training (PROMPT) [14]—were conducted in dedicated simulation suites. In situ simulations were conducted in the birth suite, ward and operating theatre. In 2020 the unit had monthly embedded multidisciplinary maternity simulations, 6 full day courses and 7 ward based simulations.

### Study process

**Fig. 2:**
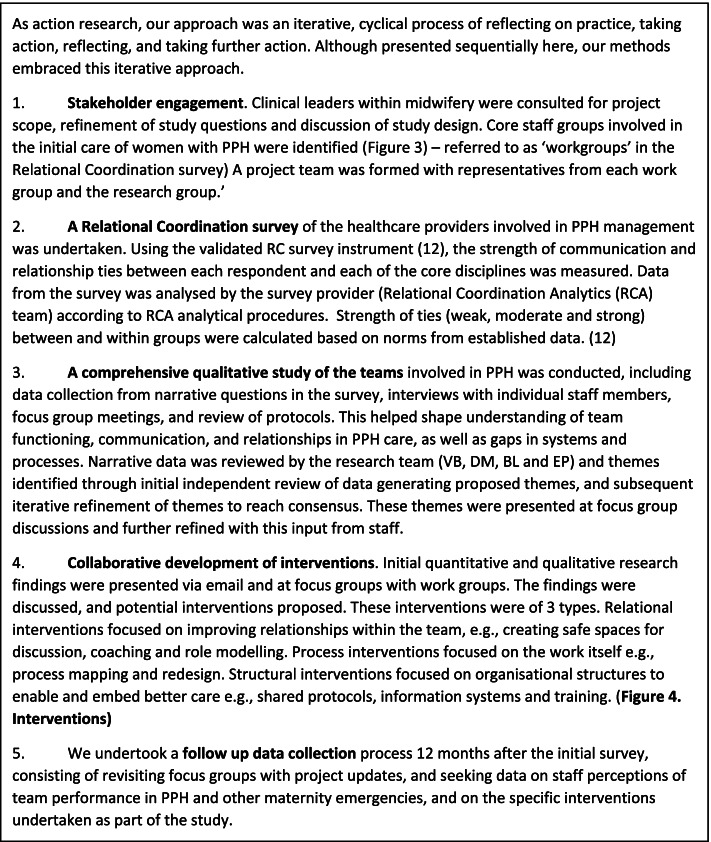
Study methods

**Fig. 3:**
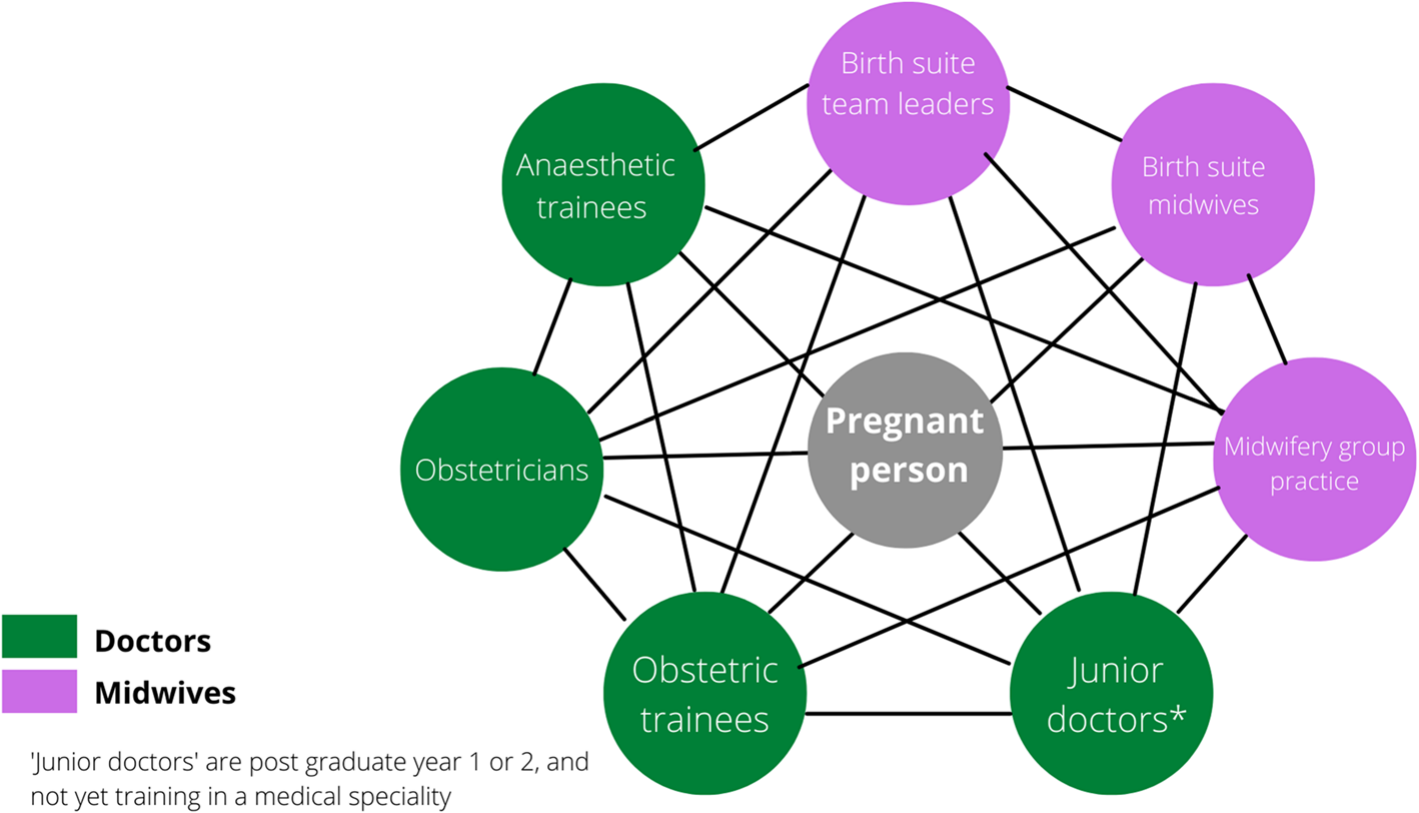
Relational coordination networks among workgroups involved in PP care

**Fig. 4:**
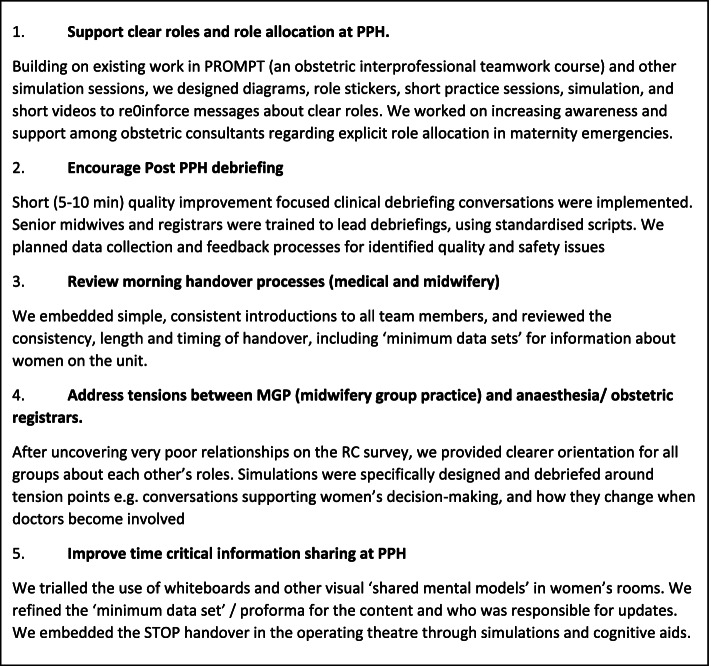
Interventions collaboratively designed and delivered with clinical teams

As action research, our approach was an iterative, cyclical process of reflecting on practice, taking action, reflecting, and taking further action. Although presented sequentially in Fig. 2, our methods embraced this iterative approach. We identified core staff groups involved in the initial care of women with PPH (Fig. 3)—referred to as ‘workgroups’ – and collected quantitative and narrative data via surveys, focus groups and during team-based simulations. In collaboration with these clinical teams, we explored systems, processes, relationships and culture relevant to the care of women who suffered a PPH. We then co-designed and implemented key structural, process and relational interventions to improve teamwork in PPH cases (Fig. 4.).

The Relational Model of Organisation Change [15]—a derivative of Relational Coordination—proposes that three types of interventions are required to support positive change. Relational interventions help build respect and support effective communication. Relational interventions include creating safe spaces for reflection, sharing, and thinking about new ways to interrelate. Process interventions enable people to apply new dynamics to the work itself. Process interventions include assessing the current state, identifying the desired state, and experimenting with solutions to close the gap between the two. Structural interventions target organisational structures that embed relational dynamics and process. Structural interventions include selecting and training for teamwork, shared accountability, and shared information systems. According to the model, the three types of interventions are expected to work synergistically to support changes in role relationships [15].

## Results

### RC survey (quantitative data analysis)

Relational Coordination (RC) Survey Response Rates**.** The overall response rate to the survey was 41% (104/253 invited participants), with participation by different workgroups ranging from 61% (midwife tea leaders) to 25% (midwifery group practice midwives).

### RC survey results: The seven dimensions: RC team score

**Fig. 5:**
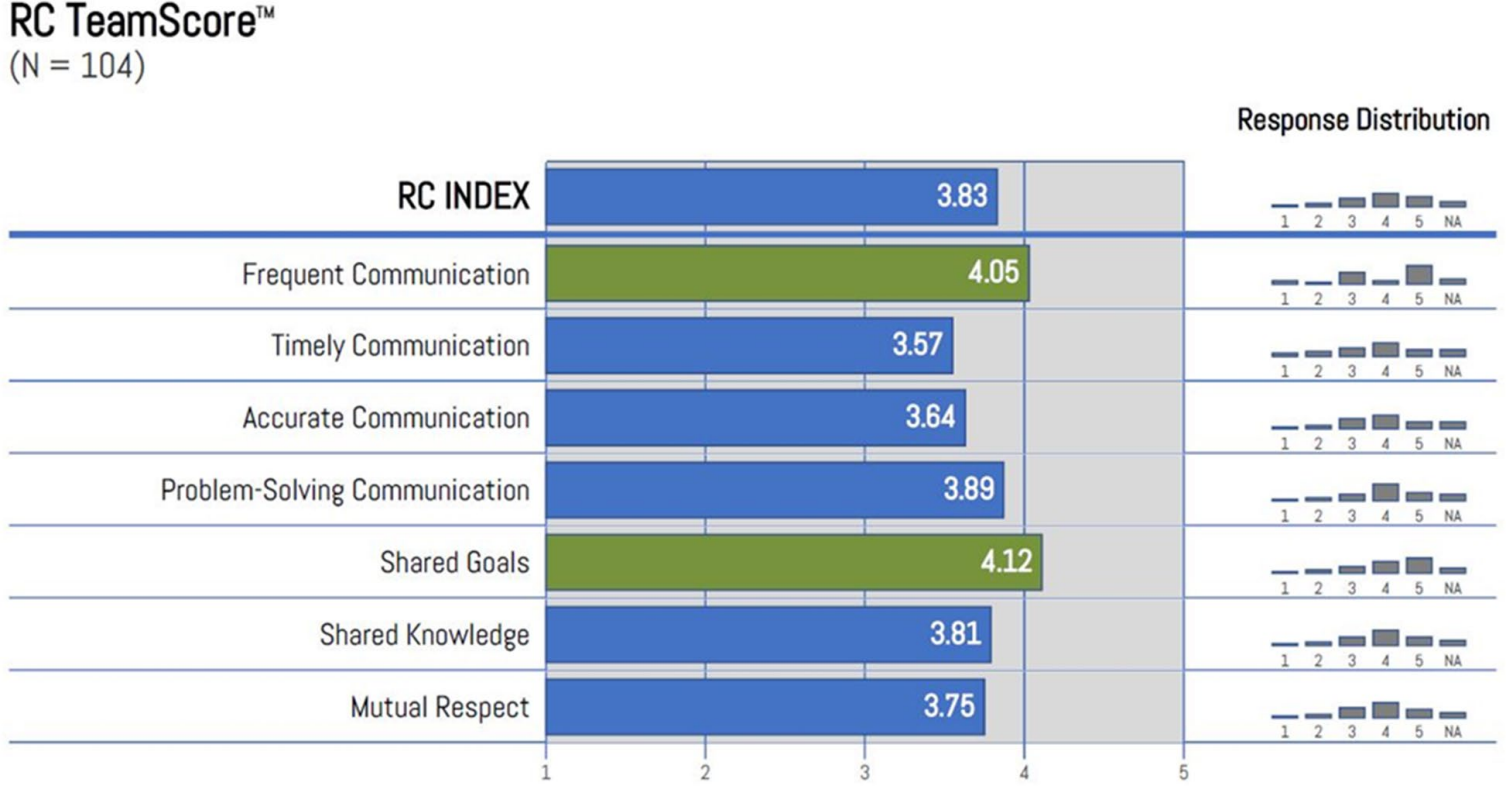
Relational coordination team score

The RC Team Score shows the overall levels of Relational Coordination for the team involved in the management of a PPH (Fig. 5). This index is an aggregate score of the seven dimensions and indicates the overall strength of Relational Coordination across all groups. Our findings suggested perceived strengths in shared goals and frequent communication, but lower scores in mutual respect, shared knowledge and in the accuracy and timeliness of communication.

**Fig. 6:**
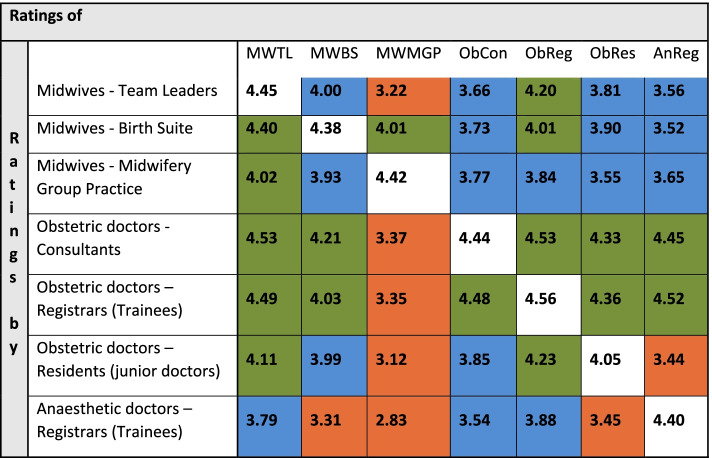
RC matrix Maternity (PPH) Relational coordination survey. The Relational Coordination Matrix shows how each workgroup rates the others (horizontal) and how it is rated by the others (vertical). E.g., to see how Midwives -Birth Suite group rated the other groups, look across the second line in the matrix. To see how the Midwives -Birth Suite group were rated by other groups, look down the second column in the matrix (MWBS). The diagonal shows how each workgroup rates itself. Between groups scores over 4.0 are considered strong, 3.5 – 4.0 are moderate, and less than 3.5 is weak. Within groups, greater than 4.6 is strong, 4.1- 4.6 is moderate and less than 4.1 is weak

Relational Coordination Matrix (Fig. 6) shows how each workgroup rates the others and how it is rated by the others. There were strong ties within groups, e.g. the obstetric registrars perceived good relationships with other obstetric registrars. There were also strong ties between some groups, such as those between obstetric consultants and midwifery team leaders. However, the matrix also identified many areas of problematic relationships, including the midwifery group practice (MGP) midwives’ relationships with almost all other groups.

### Narrative survey and focus groups (Qualitative data analysis)

We analysed 104 narrative survey responses and fieldnotes from 14 focus group meetings conducted at various stages of the project and identified five main themes – teamwork and clear roles, relational factors, identifying and managing conflict, using simulation as a reflexive tool, and optimising call systems.

### Theme 1. Teamwork, clear roles and identified leadership is critical for effective PPH management

Clarity of roles was a pervasive theme when staff were asked what happened when a PPH went well. More granular review revealed that this concept represented two processes. Firstly, a shared conception of the required roles, including the words used to name and describe them. Second, the process by which roles were allocated to team members, including pre-determined allocations (on a shift or by profession) and ad hoc allocation at the time of the emergency.

I often take on the management of “person power” and ensure delegation to the helper midwives so that multiple roles aren’t completed by the same person with roles left vacant. *[survey respondent, Midwife]*

Explicit identification of a leader(s) was highly valued during a PPH, but frequently lacking in respondents’ experience. Positive leader behaviours were focused on the clarity of decision-making and communication, especially ‘recaps’ to update on patient conditions and plans.

There are times when rooms are just messy and chaotic—and I think this happens when there's not a clearly defined leader or leaders, when the team is confused about what management has or hasn't happened or when there hasn't been recognition of the gravity of the situation. *[survey respondent midwife]*

There was a tension between the leader role of setting strategy i.e., deciding on what management was required, and managing the team to execute that strategy. Participants cited examples of a ‘good PPH’ where the burden of these two roles was shared in a co-leadership model.

I liaise with the doctors to ensure they have the right help they need and ensure recapping so that the right level of care is being received. [survey respondent midwifery team leader]

**Fig. 7:**
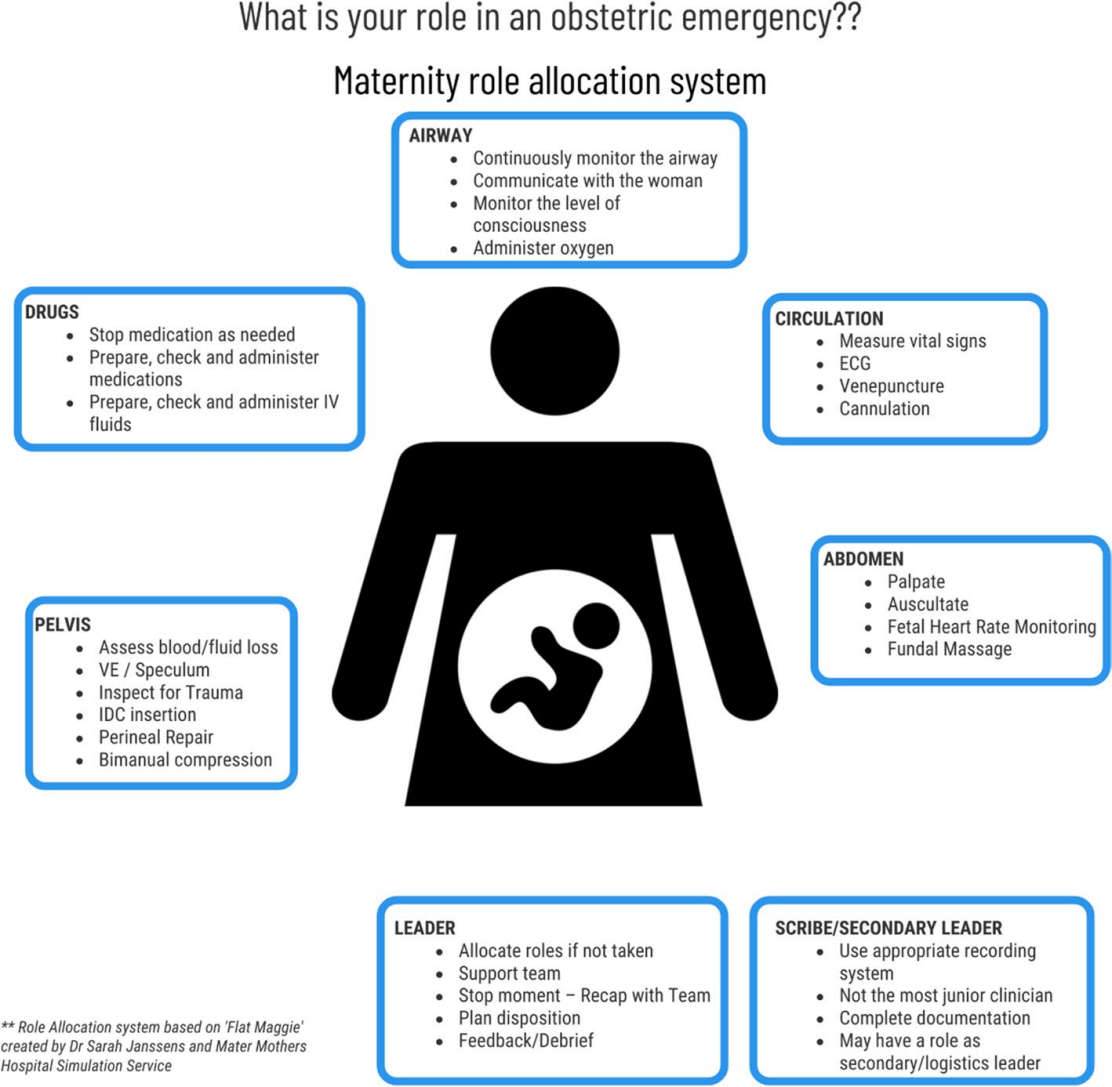
Maternity role allocation cognitive aid

We implemented enhanced multidisciplinary team training as a core intervention. Role descriptions and visual aids for maternity emergencies were developed (Fig. 7). The maternity emergencies course was revised, with a renewed focus on roles, role allocation and team leader communication. We increased the number of multidisciplinary in situ simulations delivered in birth suite and targeted our debriefings to discussion of these teamwork issues. ‘Mini-sims’—5 min role play scenarios—were conducted in education sessions and handovers to reinforce role allocation.

These interventions were perceived as overwhelmingly positive during focus group discussions one year after project commencement. The team continues to identify ways to embed high frequency, short simulations into daily practice. Of interest, the best examples of real world care were often when a simulation facilitator/ project subgroup leader was leading the team, suggesting that deep and frequent experience leads to high performance.

### Theme 2. Relational factors underpin teamwork behaviours—Shared goals, shared knowledge, and mutual respect

The descriptions of overt, observable teamwork behaviours during a PPH emergency were often complemented by descriptions of ‘feeling’ in the room as a differentiator between a ‘good’ and ‘bad’ PPH. While often ascribed to the tone set by leader behaviours, the team affect was also profoundly affected by perceptions of trust and relationships. Respect was explicitly mentioned numerous times in survey responses and focus groups and across professional disciplines.

“[ when a PPH goes well] *everyone is calm, everyone in the team knows what is going on, people are not getting angry at each other (if they disagree or challenge, it is in a polite and respectful way), it feels like everyone trusts everyone, there is lots of check-in and check-back (especially between obs and anaesthetics). The registrar feels free to mention something to a consultant that they think might be helpful.” [survey respondent, obstetric registrar]*

Suggestions for improvements included more opportunities for reflective practice:

“I think we need to spend more time debriefing after incidents like PPH. Or if having a review of the way the PPH was managed. We often do this between TL/CL/Reg [team leader, clinical lead, obstetric trainee] in the fish bowl [handover room]. But I’m not sure this always gets filtered to the other members of the team eg Primary MW [midwife]. We often so busy that these simple things get missed” [survey respondent, midwife].

### Theme 3. Conflict and poor relationships should be actively explored and addressed

The baseline measures of Relational Coordination revealed some troubling tension points. Two specific examples are illustrative.

Firstly, we heard that one group – anaesthetic registrars – felt excluded from the birth suite team. Some of this sentiment maybe due to their positioning as a relative outsider in the birthing process, as compared to midwives and obstetricians, and the relative infrequency of their rostering to birth suite among their other anaesthetic lists. However, we discovered a process issue in their lack of inclusion in core morning handover, with one anaesthetic registrar saying that he “*felt like a door stop*”, as anaesthetic registrars often literally stood at the doorway, unintroduced, while the handover was conducted.

“They [anaesthetic registrars] feel left out of handovers in the morning and evening—no dedicated time/ opportunity to either ask or hear for their perspective, and they just listen in and others assume they’ll work out what needs doing. They would like a ‘liaison point’ for efficiency in birth suite—i.e. not being contacted by multiple people but rather have a holistic prioritisation of their contribution” [field notes, anaesthetic registrar focus group].

Other workgroups were surprised and dismayed to hear the sentiment and undertook a significant revision of the morning handover location, physical environment, and process. This had immediate positive impact on those involved in handover, and at the 12 month follow up we noted a very different relationship with anaesthesia that extended beyond handover.

“Communication is better – more frequent and timely. TL talking with Anaesth Reg at start of shift and throughout when needed- this is helpful. Recognized that TLs are helpful in mitigating the problem of less experienced midwife calling the anaesthetic registrar too often/unnecessarily, and this approach should be encouraged more”. [Follow up focus group Anaesthetic registrars].

“The biggest thing for me personally is that simulation has meant I have got to know a lot of the perioperative and anaesthetic staff-and I feel like these relationships has helped me tenfold with so many other elective and emergency cases for the real patients. It's so much easier working with people you know and trust.” [Follow up email correspondence obstetric registrar].

The second example was the RC survey results indicating weak ties between the midwifery group practice midwives (MGP) and most other groups. The lower survey response rate of the MGP group also signalled this disaffection. Some tension points were obvious to those involved in women birthing within the MGP practice model – differing perspectives on birthing interventions between the women, the midwifery groups and the medical groups. What was less obvious was how these perspectives could be reconciled by a mutual respectful team into a plan that balanced women’s autonomy with a ‘medical’ focus on safety. The emotional impact of mutual disrespect was often unrecognised.

“With the midwifery group practise anaesthetic registrars feel “disrespected” and feel as though they are merely a service to be ‘ticked’. In making sense of this, one thought was that this behaviour could reflect the MGP midwives coming to terms with a “failure of the birth plan” rather than a lack of respect for the anaesthetists as such.” [fieldnotes, anaesthetic registrar focus group].

Given the intensity of this issue, our team felt that simple simulation sessions involving PPH care were inadequate to deal with the conflicting perspectives, and risked being tone deaf to the gravity of underlying resentment. Instead, we designed a bespoke session that involved facilitated discussion, triggered by a video drawn from the television program “One born every minute’. Multidisciplinary attendees offered insights into the thinking underneath the observed behaviours seen among caregivers attending to a couple who were planning a VBAC. This had a powerful impact on the groups involved, and was reflected in a high level of engagement of the MGP midwives in the improvement process.

“We often have these type of discussions in an ad—hoc manner but to have it as a structured conversation and in a multidisciplinary setting was very beneficial. It challenged us to think about it from others’ perspectives, and allowed me gain insight into what was important to some people that I hadn’t necessarily been as aware of. It was also interesting that a few people’s personal experience of thing that happen to themselves has clearly had a significant impact on them and how they practice.” [Obstetrics senior registrar].

The MGP and medical teams have committed to ongoing multi-disciplinary education and simulation exercises. Trainees from both anaesthesia and obstetrics had fewer concerns with the relationship in discussion in the 12 month follow up focus groups, and MGP midwifery group had suggestions for new areas of practice to focus on.

### Theme 4. Simulation supports better team performance, by multifaceted mechanisms

Simulation exercises were well regarded both prior to and during the project. Simulation was viewed as a method to practice knowledge and skills, and to enhance teamwork behaviours.

The research team prompted revisions and additions to simulation formats, including more ‘mini-simulations’ and mental rehearsals, a renewed focus on role and role allocation within the SMART course, and a renewed commitment to multidisciplinary simulations, in both birth suite and operating theatre. Workshops have also been conducted for midwifery team leaders on clinical event debriefing, based on demand from staff, and leaders’ perceptions that this may help with both defusing situations of high emotion, and provide an opportunity for team-based reflection on performance.

Although perceived by the staff groups as a primarily educational tool, the research and faculty groups came to appreciate a deeper impact – as a reflexive tool for exploring and improving the work of teams [16], and as an important lens into the psychological safety of maternity teams [17].

### Theme 5. Call systems and communication aids are effective when closely aligned to their purpose

GCUH has a 2-tiered response for post-partum haemorrhage. A ‘PPH Alert’ is triggered by 800 ml estimated blood loss (EBL) and notifies a local team of midwives and obstetric and anaesthetic doctors. A ‘PPH Respond’ is triggered by more than 1500 ml blood loss and notifying operating theatre and medical emergency teams.

Although designed as a safety initiative, we found that the automatic notification of obstetric consultants with PPH alerts was having a paradoxical effect, with registrars reluctant to call an alert, knowing that the situation at hand was unlikely to require assistance from consultants but may “bother” their more senior staff. At worst it created annoyance and perception that the signal to noise was wrong.

"Stop notifying consultants that there might be a problem (ie. PPH alert calls). We have faith in our registrars and midwives and don't need to know about every possible/maybe/if the moon turns blue type scenario.’ [survey, obstetric consultant].

So a surprisingly simple intervention was to remove obstetric consultants from the PPH alert call.

Taking the consultant off the call has been positive. Now more likely to call an 'alert', because won't get (disruptive) phone call from consultant. One of the registrars mentioned essentially not using the calls before and now uses them frequently to "get the resources I need but not more". [Follow up focus group, obstetric registrars].

**Fig. 8:**
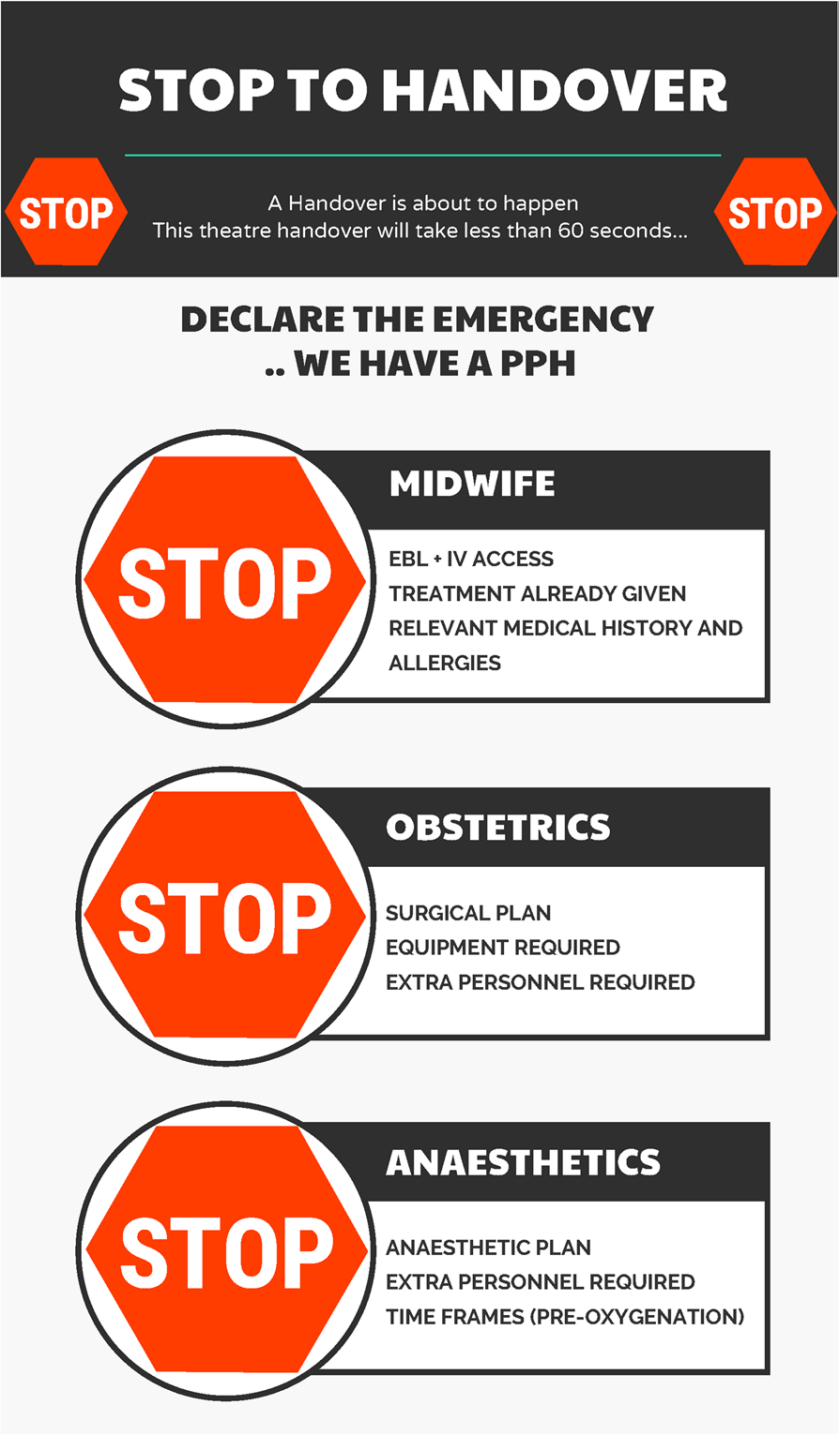
STOP to handover

The handover in theatre when an unwell woman arrives from birth suite for operative intervention is a critical transition moment requiring succinct and meaningful communication from one team to another. The STOP handover process and infographic (Fig. 8) was designed to support that process, and has been tested and embedded in simulation.

### Clinical outcomes

**Fig. 9:**
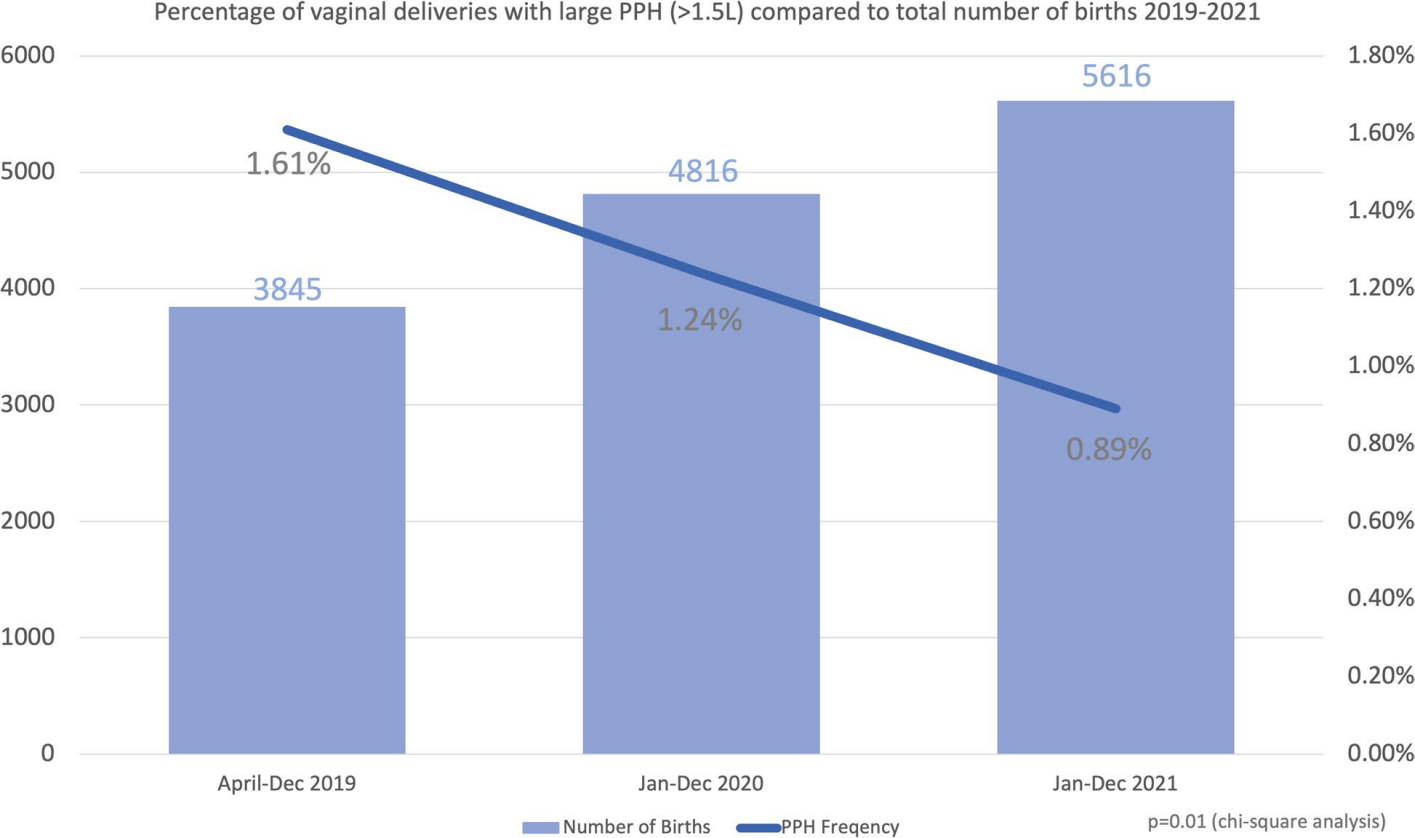
Large PPH versus total births

Effective early management of a PPH is a major influence on whether the situation progresses to a large PPH (> 1.5L). Therefore we used the number of large PPHs(> 1.5 l) as one indicator of the quality of care provided to women suffering a PPH at our institution. From April 2019 – December 2021 there were 172 women who had a vaginal birth with a PPH > 1.5L. Trends in total number of births and percentage of vaginal deliveries who progress to a large PPH is shown in **Fig. ****9****.** Despite an increase in the number of births over the study period, there was decrease in the number of women with large PPH (chi square analysis (p < 0.01). Furthermore, there was no increase in transfusion requirements or number of transfers to the operating theatre.

## Discussion

### Context shaping, over quality improving

In keeping with maternity and resuscitation literature, we found that teamwork, relationships, and simulation training all contributed to team effectiveness in caring for women who suffer a PPH at our institution [3, 12, 18]. The conceptual framework of Relational Coordination gave a tangible language and shared model for both the clinical teams and the project group to understand relationships and their impact. We found that relationships and culture can be explored and shaped deliberately through a dedicated process that involves clinicians across professions and specialities. Clinicians are capable of supporting and leading self-driven, expert guided, context-improvement initiatives. Our approach of supporting everyday clinicians in structural, relational and process improvements is messier than many more linear quality-improvement projects but serves as a model that is appropriately respectful of, and responsive to, the tangled social-technical realities of providing care in birth units and other complex environments. Some may see this messiness as a limitation, we feel it is the greatest strength of our work. It is a move towards a safety approach that focuses on creating contexts and environments in which teams can continuously learn and grow and improve, together [4, 5, 19, 20].

### Reflections on specific context shaping approaches

An in-depth discussion of each of our approaches is outside the scope of this discussion. Here we highlight two of our most pertinent reflections.

Conflict between departments and professions is widely reported in healthcare and associated with poorer outcomes for patients [21]. In our experience, avoidance is a commonly employed, but rather dysfunctional, strategy for mitigating conflict for many clinical teams. Relational Coordination was an effective way to showcase relational issues within our groups (rather than between individuals) and we experienced that uncovering these tension points was the first step towards addressing them meaningfully. Once uncovered, conflict could then be managed directly and openly. This required a thoughtful approach with expert guidance and conversational support. Although neither a comprehensive nor singular solution, translational simulation debriefings and learning conversations allowed a psychologically safe place to discuss vulnerabilities and fears. This is in keeping with a similar experience our group has facilitated with trauma care [12].

We were surprised at how frequently small, no-cost, things could make a big difference e.g., introductions at the morning handovers, changing the PPH alert calls, and instigating short ‘mini-sim’ mental rehearsals. This aligns with the extensive literature on behaviour change [22] – that consistent, small habits are more effective than large scale interventions that lack feasibility or staff engagement. The ‘small moments matter’ principle is also consistent with our recent study findings on psychological safety in the emergency department at GCUH and our previous work in trauma care that illuminated the important of team briefings [23, 24].

### Key lessons

Our lessons for health service and clinician leaders are to support team development that is relationship and culture-based, and to actively promote values of shared goals, shared knowledge and mutual respect. Use collaborative approaches to improving quality and safety with engaged frontline clinicians. Spend time and effort understanding conflict within and between teams as the first step to working better. Focus on context improvement as much as quality improvement.

Our lessons for simulation programs are to spend time and resources understanding teams’ contexts – through simulation and other methods—before designing simulation based interventions. Recognise that simulation can do harm if badly designed or delivered. Use language with simulation participants that fosters principles of Relational Coordination (or similar frameworks) to maximise transfer of behaviours to real patient care. Expand your repertoire of team development activities beyond large scale immersive simulations so techniques can match objectives.

Our lessons for frontline clinicians are to insist on a role in improving quality and safety in maternity care and beyond. Be reassured those conversations about work relationships and culture, even if uncomfortable, matter. Embrace simulation as a technique and help guide simulation providers as to the appropriate targets for their work.

## Conclusion

Our clinical teams were highly engaged in exploring their teamwork, values and relationships, and in developing and testing better ways of doing their work. They have provided a deeper understanding of effective teamwork, strategies to address tensions, and more nuanced ways to design simulations and other team training interventions. During this time of unprecedented activity in labour ward, teams caring for birthing women have reduced the frequency of large PPHs, and report a picture of evolving teamwork, with more work to do.

## Data Availability

Interview transcripts not available due to confidentiality provisions of ethical approval. Thematic analysis and qualitative data available on reasonable request from the corresponding author.
